# Effects of energy drinks on repeated sprint performance and cognitive function in athletes

**DOI:** 10.3389/fphys.2026.1796957

**Published:** 2026-05-07

**Authors:** Li An Liao, Pin Chia Pan, Chi Hsiang Hung, Yu Jui Li, Kun Tien Wu, Jie Ping Wang, Chien Wen Hou

**Affiliations:** 1 Office of Physical Education, Soochow University, Taipei, Taiwan; 2 Laboratory of Exercise Biochemistry, Department of Sports Sciences, University of Taipei, Taipei, Taiwan; 3 Department of Ball Sports, University of Taipei, Taipei, Taiwan; 4 Department of Recreation and Sports Management, University of Taipe, Taipei, Taiwan; 5 Department of Physical Education, Shih Hsin University, Taipei, Taiwan; 6 School of Exercise and Health, Shanghai University of sport, Shanghai, China

**Keywords:** anserine, caffeine, elderberry, high-intensity exercise, sports supplements

## Abstract

**Objective:**

Energy drinks are often used by athletes before competitions to enhance their performance. Resent research has pointed out that the performance effects of select ingredients have been studied in-dividually, but not in combination with caffeine. Therefore, this study investigated the effect of caffeine, anserine and elderberry on cognitive function and repeated sprint performance of athletes during a high-intensity exercise protocol.

**Methods:**

We employed a double-blind, randomized, counter balanced, and crossover design. Three types of supplements were tested: placebo, caffeine (220mg), and energy drink (220mg caffeine, 288mg anserine, 400 mg of elderberry, and 660 mg vitamins-minerals mixed). Twelve college athletes were recruited to complete repeated sprint tests on a cycle ergometer until they reached exhaustion and undertook a series of cognitive tasks during running.

**Results:**

Results showed that energy drink group significantly improved repeated sprint performance and showed a potential to attenuate certain aspects of cognitive fatigue.

**Conclusion:**

The multi-ingredient energy drink significantly elevated total power output and attenuated the exercise-induced decline in cognitive performance. It might be a better strategy for athletes to optimize physical performance and mitigate cognitive fatigue.

**Clinical Trial Registration:**

https://clinicaltrials.gov/study/NCT07104188?intr=NCT07104188%20&rank=1, identifier NCT07104188.

## 1 Introduction

The promotion of energy drinks for enhancing performance and cognitive function in sports has garnered significant attention among athletes (Hoyte et al., 2013). As a competitive athlete, possessing sharp cognitive skills such as focus, attention, and memory is crucial for peak performance and success on the field or court. In athletic competition environments, recognizing and mitigating the factors that impairing cognitive function during sports are crucial for athletes aiming to maintain peak performance. For example, physical and mental fatigue, dehydration, insufficient sleep inadequacy, or exercise-induced fatigue are common challenges for athletes. Although there is a wide variety of supplements on the market, it is necessary to differentiate them according to scientific evidence supporting improved sports performance (Kennedy and Wightman, 2022).

Especially, repeated sprint, which is one of the most common movements in many sports, belongs to high intensity exercise. It has a high likelihood of impairing athletes’ cognitive function. The negative effects of high-intensity exercise on cognitive performance can be explained by several factors, such as the level of nervous system activation, blood acidosis, the accumulation of metabolic waste, changes in attentional strategy, or alterations in humoral function (Brisswalter et al., 2002). Previous study have also shown that exercise can lead to declines in decision-making, attention, and perceptual abilities of players (Skala and Zemková, 2022). It is important for athletes to continuously seek new information and strategies to enhance cognitive function, as this can significantly benefit their performance.

Several studies have shown that energy drinks increase subjective alertness and improve performance on attention and memory tasks as well as on tests of executive function (Emma, 2014). The primary ingredient in most energy drinks is caffeine, a well-known stimulant that can enhance alertness and reduce fatigue. In the past, the effects of caffeine or other select ingredients have been studied individually, but their combination with caffeine has not been adequately addressed. Additionally, there is limited information available about other common ingredient (Kennedy and Wightman, 2022). However, past studies revealed that anserine has been thoroughly investigated as a dietary ingredient and supplement for its potential health benefits and performance-enhancing properties. During high-intensity exercise, the ingestion of carnosine and anserine may potentially improve performance (de Jager et al., 2022). Anserine contributes to maintaining cellular equilibrium by enhancing antioxidant activity and preserving the integrity of cells in healthy men. Nonetheless, the temporary increase in exercise-induced cell damage associated with anserine, coupled with its improved antioxidant activity and hematological responses, indicates an enhanced adaptive response to exercise and better recovery (Alkhatib et al., 2020). On the other hand, elderberry is well known for its abundant flavonoids. Consuming flavonoids can improve cognitive performance through improving brain-derived neurotrophic factor (Hashimoto et al., 2004). Antioxidant supplementation may enhance performance by maintaining excitation–contraction coupling and central drive (Bowtell and Kelly, 2019). Elderberry, anserine and caffeine may have an antioxidant effect and protect cells from oxidative damage (Alkhatib et al., 2020; Liu et al., 2022; Wang et al., 2022). Theoretically, the combined efficacy of these ingredients may stem from their complementary physiological pathways. While caffeine primarily acts as a stimulant to enhance alertness, anserine provides a critical biochemical defense; its three ionizable groups allow it to sequester protons and regulate acid-base balance during high-intensity exercise (Suzuki et al., 2006). Furthermore, anserine and the flavonoids in elderberry may provide synergistic antioxidant support, preserve cerebral blood flow and stabilize biological membranes against exercise-induced oxidative stress (Hisatsune et al., 2016). Past research has focused on both combination products and their caffeine content alone and/or the combination minus caffeine (Jiménez-Alfageme et al., 2022).

Given that most existing research focuses on caffeine in isolation, it remains unclear how its effects compare to those of combination products such as energy drinks (Kennedy and Wightman, 2022). The purpose of this study is to compare the effects of caffeine alone and a combination energy drink (caffeine, anserine, and elderberry) on cognitive function and exercise performance, with particular attention to the contribution of non-caffeine ingredients. The research question is how do caffeine alone and a combination energy drink differentially affect cognitive function and exercise performance? Our primary hypothesis was that the combined ingredients (caffeine, anserine, and elderberry) would yield superior cognitive benefits compared to caffeine alone. An energy drink containing caffeine, anserine, and elderberry will produce greater improvements in cognitive function compared to caffeine alone.

## 2 Materials and methods

### 2.1 Participants

The study population was selected by healthy male college athletes of wrestle (173.27 ± 2.31cm; weight, 75.36 ± 3.31kg; age, 21.45 ± 1.56 year). People who are sensitive to caffeine were excluded. Twelve college wrestlers participated in this experiment. The sample size was determined using G*Power 3.1.9.7. For a one-way repeated measures ANOVA, assuming a medium-to-large effect size (ƒ=0.435), an alpha level of.05, and a power of.8 with three measurements, a minimum of 12 participants was required to detect significant differences between conditions. Participants were asked not to change their dietary habits during the experiment, be-sides, they were also asked to maintain their training frequency. In order to eliminate the mixed effects of caffeine withdrawal symptoms, participants were required to quit caffeine or caffeine-containing products within 48 hours before the experiment, and not to do any strenuous exercise. All participants gave their written informed consent. The first participant was enrolled on 2022 May 10th, and the final follow-up assessment for primary outcomes and safety monitoring was completed on 2022 June 16th. The total duration of the study period, including the two treatment phases and the washout period, spanned approximately 5 weeks. The trial reached its planned conclusion after all enrolled participants completed the follow-up period. No interim analyses or predefined stopping guidelines were implemented for this study. Standard ethical procedures remained in place, allowing for the immediate cessation of a session if a participant requested to withdraw or experienced discomfort.

### 2.2 Study design

This study employed a double-blind, randomized, counter-balanced and crossover research design, in which all 12 participants completed all three intervention conditions. To minimize potential order effects, participants were randomly assigned to one of three treatment sequences treatment sequences (e.g., Sequence A, B, or C). Both the participants and the primary investigator remained unaware of the treatment assignments throughout the trial. The trial was conducted at a university in Taiwan Taipei Shilin district. All participants were divided into three groups to complete the counterbalanced test. They needed to visit seven times, separated by a one-week washout period. These tests were conducted at a similar time of the day, with controlled temperature. During the familiarization visit, body height, body mass, and seat height were recorded. The primary investigator (PI) and trained research assistants administered the tests. This study was approved by the Institutional Ethics Committee of the University of Taipei (IRB-2021-021), and all procedures conformed to the Helsinki Declaration for the re-search involvement of human participants.

**Figure 1:**
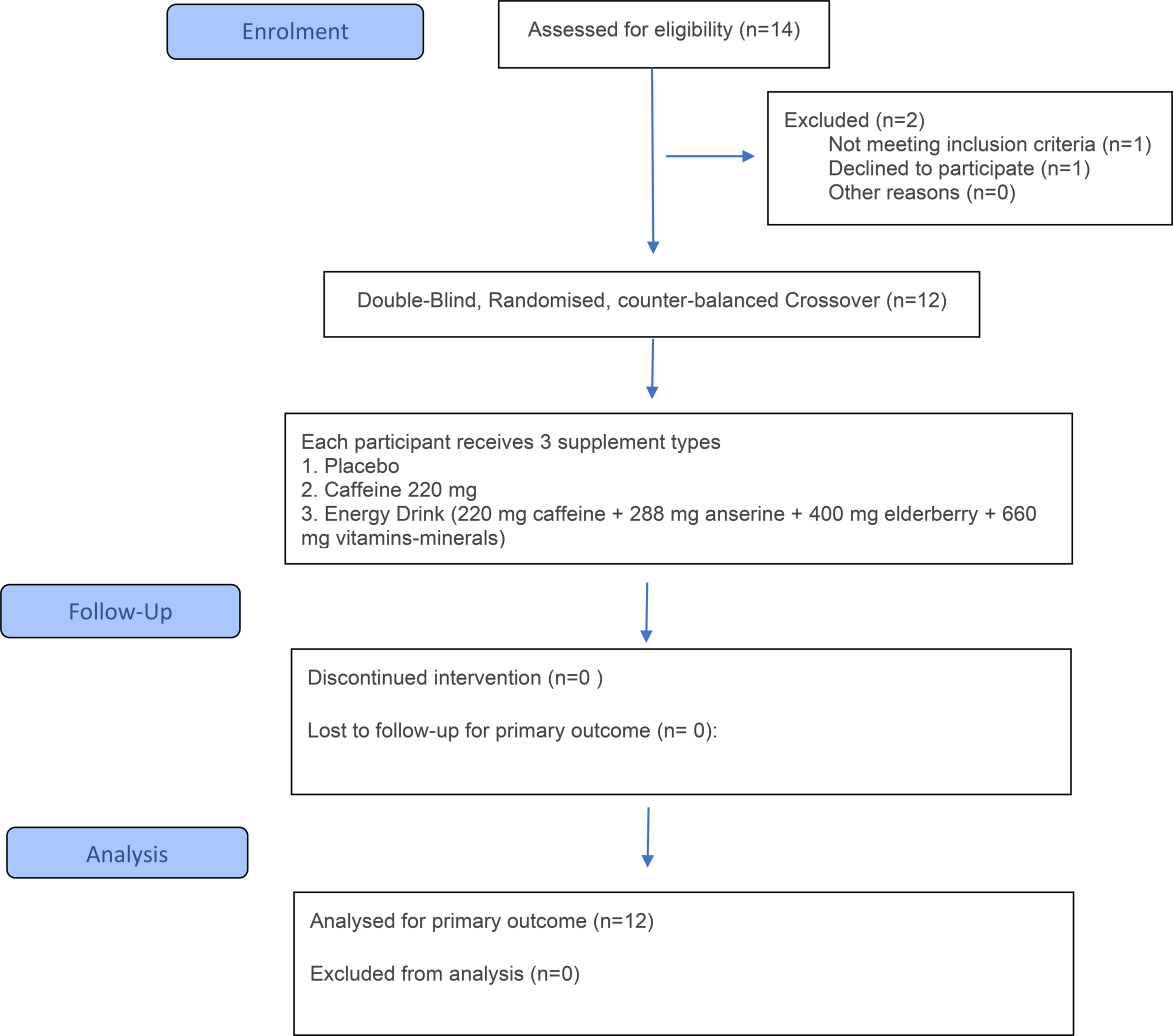
CONSORT flow diagram.

The flow of participants through the study is summarized in **Figure 1** total of 14 participants were screened, and 12 were ultimately enrolled and completed.

### 2.3 Instruments and procedures

#### 2.3.1 Supplement

There are three kinds of supplements: 1) placebo; 2) 220 mg caffeine; 3) 300 ml water mixed with 6 g of the Energy Drink powder, which contained 220 mg of caffeine and 1348 mg of other energy matrix (288mg anserine, 400 mg of elderberry, and 660 mg v of a vitamins-minerals mix). To make sure the participants could not figure out the differences, these powders were mixed into 100ml grape juice and 200ml water., these powders were mixed into 100ml grape juice and 200ml water. Every 100ml of grape juice contained 15.6 g of carbohydrate and 0.6g of protein. Participants needed to finish the supplement 30 minutes before the tests began. During the entire test period, participants were restricted from consuming any food or water.

#### 2.3.2 Repeated sprint

After having the supplements and waiting 30 minutes, participants completed a repeated sprint test. It began in a 5-minute warm-up with 60W at a cadence of 60rpm. Then, participants began 10-second all-out maximal sprints and 20-second passive recovery until fatigue, defined as cadence < 70 rpm for more than 3 seconds. The resistance was set at 0.8Nm per kilogram of body weight (Willis et al., 2017). During the passive recovery, participants were restricted from consuming water or standing up. During the repeated sprint test, participants were given very strong verbal encouragement and were not given any hints about the number of sprints performed. We asked participants to sprint as fast as they can, besides, and as many times as they can. After finishing the repeated sprint test, we would record the total sprints, total power output and average power output. These data were used to determine the effects of different supplements on repeated sprint performance. Harms were assessed non-systematically based on spontaneous reporting by participants. Throughout the study, participants were instructed to self-report any discomfort or adverse symptoms to the researchers immediately, and no harms, or unintended physiological responses were reported by participants or observed by the investigators during any of the experimental trials.

#### 2.3.3 Cognitive function tests

Cognitive function tests were tested using an iPad, and they consisted of Squares, Color Drop, and Schulte Grid. After pre-cognitive function tests, participants were asked to consume supplements and waited for 30 minutes. After that, participants needed to finish running on the treadmill at increasing speeds and grades. The exercise consisted of three continuous stages, each 3 minutes in duration: Stage 1, 4.0 km at 12% grade; Stage 2, 5.4 km at 14% grade; Stage 3, 6.7 km at 16% grade. Each participant was asked to do the post-cognitive function tests during running during Stage 3.

Squares This kind of test is used to assess spatial working memory, which requires retention and manipulation of visuospatial information (Nagamatsu et al., 2013). The spatial memory task required participants to recall the spatial location of dots presented on a screen. A 6×6 grid was shown. First, some squares were colored for one second, and after they re-turned to normal, we need to press the squares that were previously colored. It would end if participants answered incorrectly. At higher stages, more squares were randomly colored. Reaching higher stages indicates better memory performance.

Colour drop This kind of test is widely used in a variety of populations. It is a test used to assess executive function. Executive function is related to thinking and decision-making (Scarpina and Tagini, 2017). The rules involve Chinese words written in different colors. For example, the word “GREEN” is printed in blue ink, the word “RED” in yellow ink. And participants need to answer the ink color rather than the word itself. The test ends if participants respond incorrectly. Higher scores indicate better executive function.

Schulte grid This kind of test is used to assess attention. Attention is the skill of focusing on relevant aspects while ignoring distractions (Maksimenko et al., 2018). The schulte grid con-sists of 6 x 6 randomly arranged numbers from 1 to 36. Participants were asked to find numbers in ascending order. First, they had to find 1, then 2, until 36. Completing the test more quickly indicates better attention.

### 2.4 Procedure

**Figure 2:**
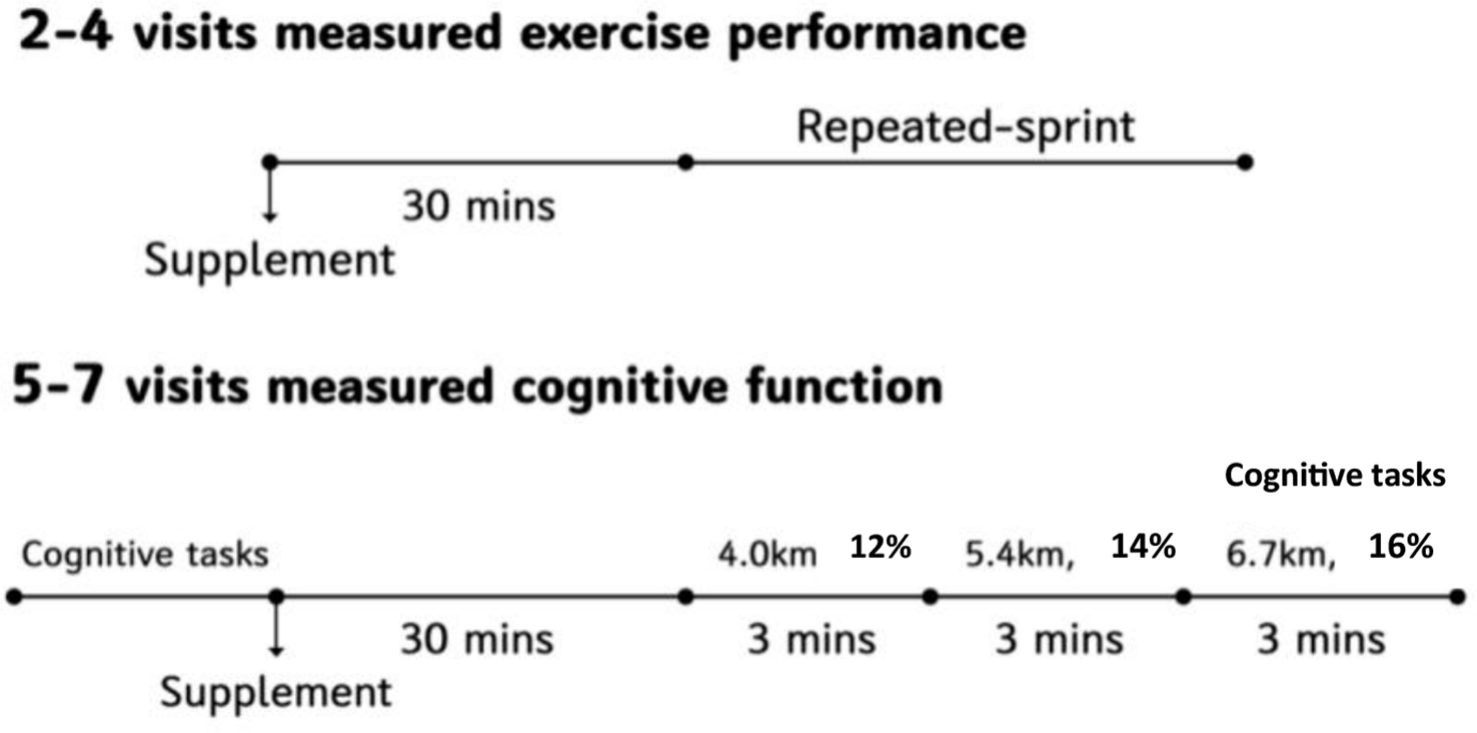
Schematic representation of the experiment schedule.

As shown in **Figure 2**, during the 2-4 visits, participants consumed the supplement, waited 30 minutes, and then complete repeated-sprint test. During the 5-7 visits, participants completed pre-cognitive function tests, consumed supplements, waited 30 minutes, and then completed post-cognitive function tests during running.

### 2.5 Statistical analysis

All data were presented as mean ± standard error (SE). All 12 participants who were randomized completed all three intervention periods. There were no losses to follow-up or exclusions after randomization. Exercise and cognitive performance data were analyzed using one-way repeated measures ANOVA to evaluate differences between conditions. Planned paired-samples t-tests with Bonferroni correction were conducted to examine pre-to-post changes within each condition. Statistical differences were set at *p* <.05. To address the assumption of sphericity for the within-subjects factors, Mauchly’s test was performed. In cases where the sphericity assumption was violated, the Greenhouse-Geisser correction was applied to adjust the degrees of freedom and p-values. No other analyses, including subgroup or sensitivity analyses, were performed. Effect sizes were reported and were defined as large (*d*>0.8), moderate (0.5–0.8), and small (<0.5) (Cohen,2013).

## 3 Results

### 3.1 Exercise performance outcomes

**Figure 3:**
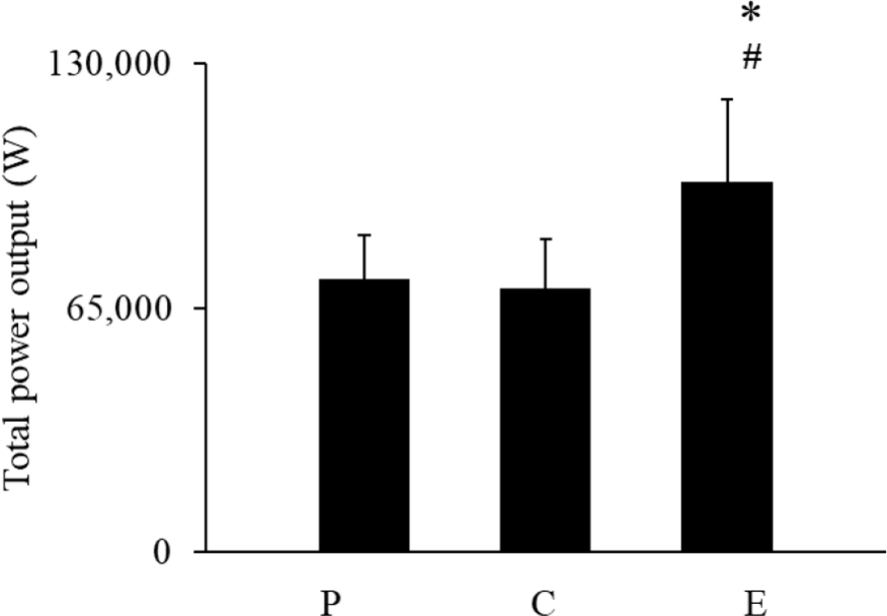
Total power output during repeated sprint performance. Data are mean ± SE. * represents *p* < 0.05 difference from P; ^#^represents *p* < 0.05 difference from C. P, Placebo group, C, Caffeine group, and E, energy drink group.

While no significant difference was found between the placebo and caffeine groups, the energy drink group showed a significant increase in total power output compared to both., *F* (*1.16,12.82*) = 6.31, *p* = .02, *η_p_^2^* = .37 (**Figure 3**). *Post-hoc* comparisons revealed that while no significant difference was found between the placebo and caffeine groups, the energy drink group showed a significant increase in total power output compared to both.

### 3.2 Cognitive function outcomes

#### 3.2.1 Squares performance- spatial working memory

**Figure 4:**
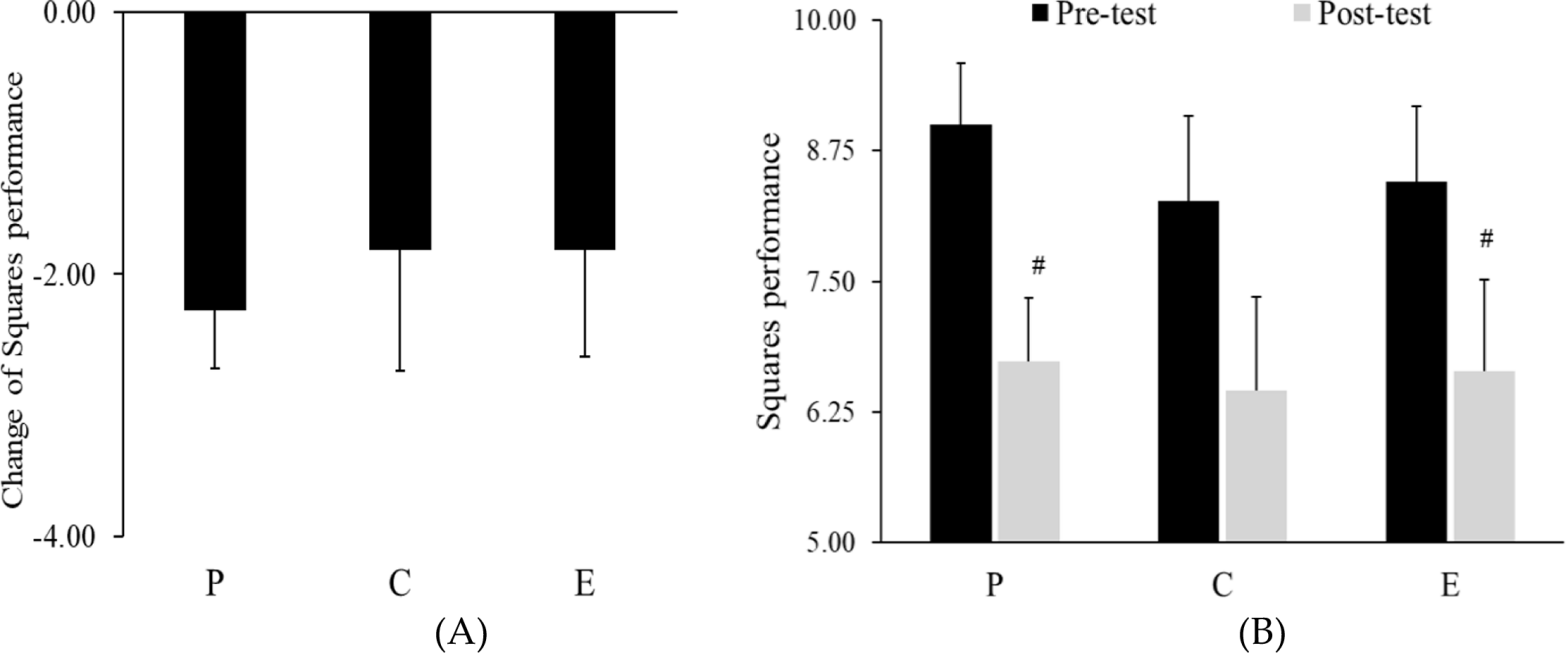
**(A)** Change in squares performance (calculated as post-test minus pre-test) and **(B)** Squares performance across groups. Data are mean ± SE. ^#^ represents *p* < 0.05 difference from pre-test. P, Placebo group; C, Caffeine group, and E, energy drink group.

Regarding Squares performance, a one-way repeated measures ANOVA revealed a significant main effect of time. Wilk’s Lambda =.299, *p* <.05, *F*(*1,11*)=25.741, *η_p_^2^* = .701. There was no significant difference between placebo group, caffeine group and energy drink group in pre-test *p*>.05 (during running performance - before supplementation) (**Figure 4A**). However, follow-up paired-samples *t*-tests, Performance in the placebo, *t*(11)=7, *p* <.001, *d* = 2.02) and energy drink *t*(11)=3.11, *p* <.05, *d* = 0.89) (**Table 1**) groups significantly declined from pre-test to post-test. (**Figure 4B**). No significant changes were found in the caffeine group”.

#### 3.2.2 Colour drop performance- executive function

**Figure 5:**
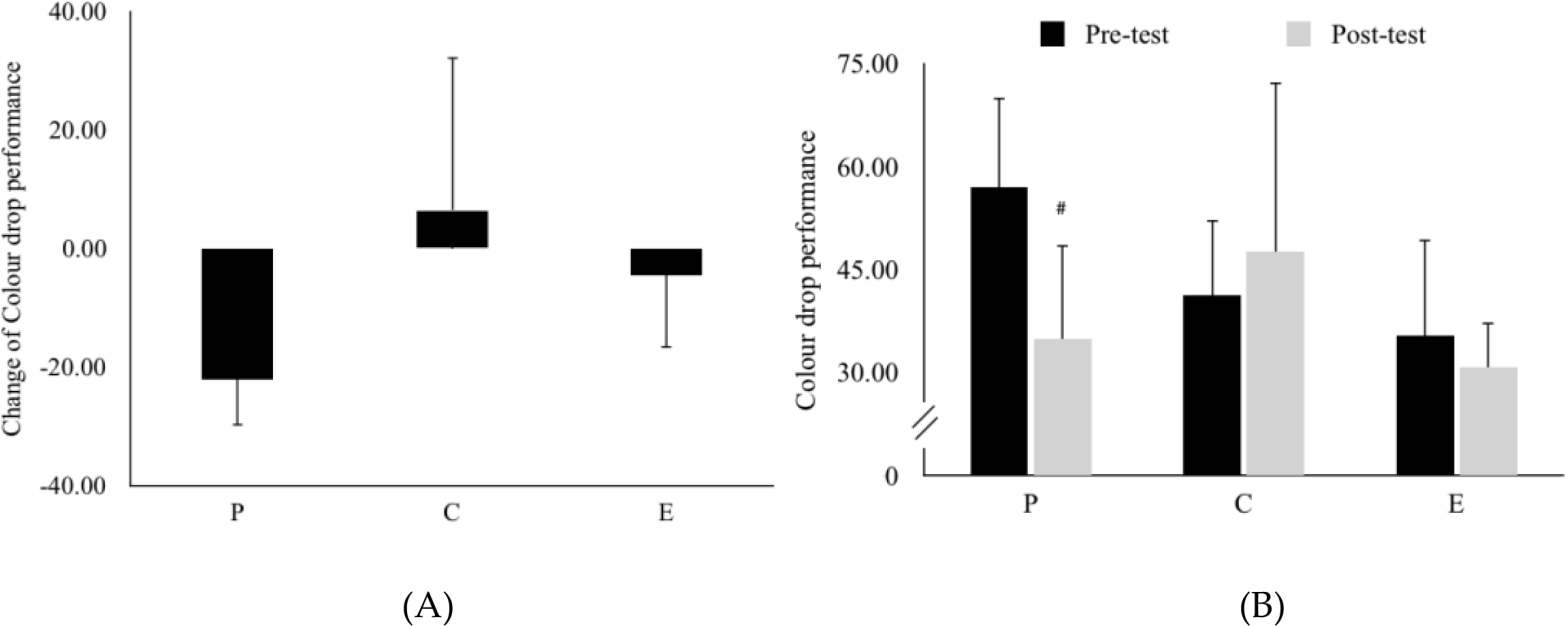
**(A)** Change in colour drop performance (calculated as post-test minus pre-test) and **(B)** Colour drop performance across groups. Data are mean ± SE. ^#^represents *p* < 0.05 difference from pre-test. P, Placebo group; C, Caffeine group, and E, energy drink group.

**Figure 5A** presented the change of Colour drop performance (During running performance - Before supplementation). There was no significant difference between placebo group, caffeine group and energy drink group. Mauchly’s test indicated that the assumption of sphericity had been violated *X^2^ (2*)=9.63 *p* <.05; therefore, Greenhouse-Geisser corrected degrees of freedom were used. *F*(*1.23,13.59*)=.1.14, *η_p_^2^* = .09. **Figure 5B** showed the Colour drop pre-to-post performance. The result revealed that only placebo group showed a significant decline from pre-test(*M* = 55.25, *SD* = 33.44) to the post-test (*M* = 38, *SD* = 36.15), *t*(11)=2.32, *p* = .041, *d* = 0.67), representing a medium-to-large effect size. In contrast, no significant differences were found in the caffeine group (pre *M* = 42.5, *SD* = 28.26, post *M* = 48, *SD* = 62.42 *p* = .77) or the energy drink group (pre *M* = 40.75, *SD* = 40.14, post *M* = 37.83, *SD* = 29.22, *p* = .75) (**Table 1**).

#### 3.2.3 Schulte grid performance- attention

**Figure 6:**
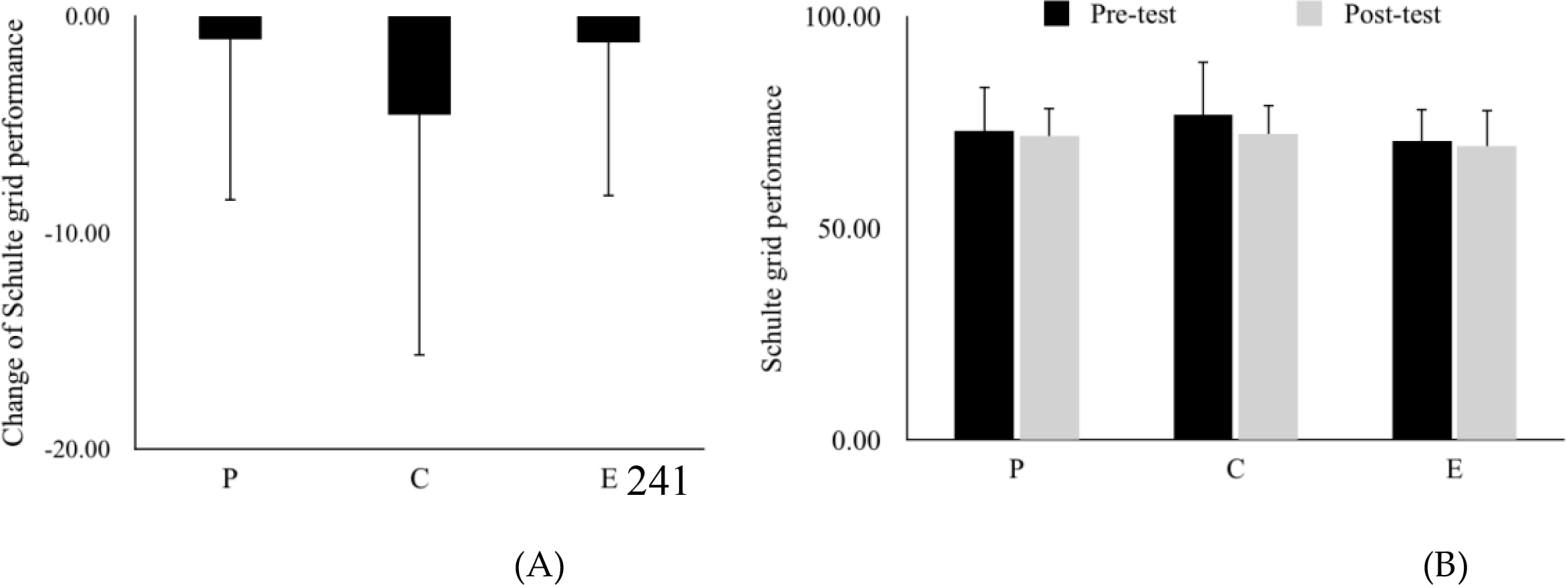
**(A)** Change in schulte grid performance (calculated as post-test minus pre-test) and **(B)** Schulte grid performance across groups. Data are presented as mean ± SE. P, Placebo group; C, Caffeine group; E, Energy drink group.

**Figure 6A** presented the change of Schulte grid performance (During running performance - Before supplementation). There was no significant difference between placebo group, caffeine group and energy drink group. **Figure 6B** post-test Schulte Grid performance, and again, no significant differences were found among the three groups. (Wilk’s Lambda =.95, *F*(*2,10*)=.286, *η_p_^2^* = .054). **Figure 5B** illustrated the post-test results, where again no significant differences were observed. Planned paired-samples t-tests further confirmed that scores remained stable from pre-test to post-test in the placebo group (*p* = .92), caffeine group (*p* = .44), and energy drink group (*p* = .89) (**Table 1**), indicating that Schulte grid performance was not significantly affected by exercise or supplementation in this study. .

**Table 1:** Mean scores and statistical results of exercise and cognitive performance pre- and post-supplementation.

Variables		Placebo (N = 12),	Caffeine(N = 12),	Energy drink (N = 12),
		72,912 ± 11604.38	70,335 ± 12939.48	98,630 ± 21960.74^#^
Squares	*p-value*	.03*	.01*	
	Pre	9.08 ± 1.50	8.33 ± 2.05	8.66 ± 1.96
	Post	6.75 ± 1.54	6.83 ± 2.62	6.83 ± 2.32
Colour drop	*p-value*	.001*(*d* = 2.02)	.06	.01*(d = 0.89)
	Pre	55.25 ± 33.44	42.5 ± 28.26	40.75 ± 40.14
	Post	38.00 ± 36.15	48.00 ± 62.42	37.83 ± 29.22
Schulte grid	*p-value*	.04* (*d* = 0.67)	.77	.75
	Pre	72.03 ± 26.60	76.78 ± 32.11	70.08 ± 19.51
	Post	71.52 ± 16.63	70.21 ± 19.01	70.85 ± 22.49
	*p-value*	.92	.44	.89

Data are presented as mean ± SD. Cohen’s d was used for post-hoc comparisons (small < 0.2, medium = 0.5, large > 0.8). *Significant difference compare with pre and post (p<0.05). ^#^Significant difference (p <.05) compared to both the Placebo and Caffeine groups in total power output.

## 4 Discussion

The present study examined the effects of a multi-ingredient energy drink, caffeine alone, and placebo on repeated sprint performance and cognitive function under exercise-induced fatigue. Overall, the findings indicate that exercise influenced cognitive performance over time, whereas no statistically significant differences were observed between supplementation conditions across cognitive outcomes.

### 4.1 Exercise performance outcomes

In this study, we found that consuming an energy drink containing caffeine, anserine, and elderberry was associated with an increase in total power output, whereas caffeine alone did not yield a significant enhancement. While these results suggest a potential advantage of the combination product, the findings should be interpreted with caution given the small sample size (N = 12). The lack of significant effect for caffeine alone contrasts with some previous meta-analyses (Salinero et al., 2019), which may be attributed to the specific repeated sprint protocol or the training status of the participants. The use of a cycling-based protocol for trained wrestlers represents a non-sport-specific modality, which may have led to premature localized neuromuscular fatigue in the lower limbs. Since the participants were not specifically conditioned for repetitive high-intensity cycling, this lack of task-specific adaptation might have blunted the expected ergogenic response to caffeine alone, as the benefits of caffeine are often more pronounced in familiar exercise tasks (Karayigit et al., 2021).

The ergogenic effect of the multi-ingredient energy drink may stem from the synergistic properties of its components. While caffeine provides central nervous system stimulation, anserine acts as a potent intramuscular buffer. With a pKa of 7.01, anserine is ideally suited to sequester protons during high-intensity metabolic acidosis (Boldyrev et al., 2013). Furthermore, anserine may enhance power output by increasing calcium ion sensitivity in skeletal muscle, allowing for greater force production without additional calcium release (Dutka and Lamb, 2004; Sale et al., 2010). Complementing this, the phenols and flavonoids in elderberry may offer antioxidant support, potentially mitigating exercise-induced oxidative stress and delaying fatigue (Corr et al., 2021). Collectively, these mechanisms provide a more robust physiological framework for the observed maintenance of power output compared to caffeine in isolation.

In addition to buffering, the antioxidant properties of both anserine and the phenols found in elderberry may have further supported performance. High-intensity sprints induce significant oxidative stress; the inclusion of elderberry flavonoids may help mitigate this stress, thereby delaying fatigue and preserving muscle blood flow (Corr et al., 2021; Martinez-Negrin et al., 2022). Collectively, these findings suggest that the combination of stimulatory, buffering, and antioxidant mechanisms may be more effective in supporting repeated sprint performance than the stimulatory effect of caffeine alone.

Individual factors such as genetic polymorphisms (e.g., CYP1A2) and habitual caffeine intake were not measured in this study. While these variables are known to modulate caffeine’s effectiveness (Pickering and Kiely, 2018), their specific role in the current findings remains speculative and should be addressed in future larger-scale trials.

Caffeine enhances exercise performance through various mechanisms, including its influence on the hormonal environment (Lovallo et al., 2005). Caffeine has been shown to raise salivary testosterone levels, which helps stimulate the nervous system, leading to greater muscle strength and performance during exercise (Kraemer and Ratamess, 2005). Previous research on the effects of caffeine on repeated sprints, consisting of 30-second sprints with 30-second active recovery indicated that consuming 3 mg of caffeine per kilogram of body weight can improve repeated sprint performance by reducing the rate of accumulated fatigue (Paton et al., 2010). In addition to original research, there was a systematic review and meta-analysis indicated that acute ingestion of caffeine (from 3 to 6 mg/kg of body weight) can increase the total numbers of sprints in repeated sprint tests (Salinero et al., 2019). This benefit is not caused by reducing the rate of perceived exertion during exercise. Thus, this meta-analysis suggests that caffeine may stimulate several enhanced physical aspects related to success in sports. Moreover, it was also found that the benefits of caffeine are highly related to an individual’s regular caffeine consumption. People who regularly consume moderate-to-high amounts of caffeine (130-300 mg per day) may experience less of a performance benefit from caffeine compared to those who consume low amounts (40-50 mg per day) (Bell and McLellan, 2002). According to this study, while caffeine does not increase total power production, energy drinks do. It simply highlights how crucial having a variety of ingredients is.

### 4.2 Cognitive function outcomes

The findings of this study indicated that high-intensity exercise exerted a significant negative impact on cognitive performance, likely due to exercise-induced fatigue. However, supplementation with either caffeine or the multi-ingredient energy drink appeared to attenuate this decline in specific cognitive domains.

#### 4.2.1 Spares performance- spatial working memory

Our results showed that spatial working memory (Squares) significantly declined in the placebo group, whereas this reduction was mitigated in the caffeine and energy drink groups. While some previous research suggests that caffeine has limited effects on memory tasks (Lanini et al., 2016; Mednick et al., 2008), the preservation of performance in our study may be linked to the high cognitive demand of the running task, which allowed the stimulatory effects of the supplements to become more apparent (Smith et al., 2016).

#### 4.2.2 Colour drop performance- executive function

Executive function is essential for regulating and synchronizing cognitive activities during physical exertion. While caffeine is known to improve visual attention and executive control by acting on dopamine and adenosine pathways (Brunye et al., 2010), our data showed that only the energy drink and caffeine groups maintained relatively stable performance compared to the placebo. This suggests that the supplements may help sustain executive control even when physiological fatigue is high.

#### 4.2.3 Schulte grid performance- attention

Regarding attention, no significant differences were observed between conditions for the Schulte Grid. This contrasts with some research suggesting that caffeine enhances attention as an adenosine antagonist. The lack of a significant effect in our study might be attributed to the specific dose (220 mg) or individual differences in caffeine sensitivity. For people who regularly take large amounts of caffeine, a greater dose may be required to improve attention (Brunye et al., 2010). Lack of consuming caffeine habits investigation is one of our limitations.

### 4.4 Summary of multi-ingredient effects

The multi-ingredient energy drink’s unique profile—combining caffeine, anserine, and elderberry—may offer a broader neuroprotective effect than caffeine alone. Anserine has been suggested to support executive performance by modulating prefrontal cortex activity (Yamano et al., 2013), while elderberry phenols may support cognitive health by enhancing cerebral blood flow (Bell et al., 2015). Although we did not observe superior cognitive scores in the energy drink group compared to caffeine alone, it is noteworthy that the energy drink was the only condition associated with both elevated physical power output and a mitigated decline in cognitive performance. This dual benefit suggests that the combined ingredients may help athletes optimize physical output while concurrently buffering against the cognitive fatigue induced by high-intensity exercise.

### 4.5 Limitations

There are several limitations of the present study. First, the daily caffeine consumption habits of the participants were not formally assessed. For individuals with habitual high caffeine intake, the specific dose used in this study (220 mg) might have been insufficient to elicit maximal cognitive or physical enhancements (Brunye et al., 2010). The high inter-individual variability observed in cognitive tasks, as indicated by the large standard errors, further underscores the potential influence of participants’ habitual caffeine consumption. Such variability is common in caffeine research, where individual sensitivity and habituation can lead to diverse performance outcomes. Second, the cognitive assessments during Stage 3 were conducted while participants were running at a steep incline (16% grade). The absence of a fixed mounting system for the iPad meant that physical instability and hand-shaking could have introduced mechanical noise, potentially compromising touchscreen accuracy. Although this setup mimics the challenges of real-world cognitive-motor dual-tasking, the performance in this stage may partially reflect fine-motor interference rather than purely cognitive changes. Finally, a limitation relates to the ecological validity of the performance assessment. Although the participants were trained wrestlers, the repeated sprint test was conducted using a cycle ergometer, which may not fully reflect the sport-specific performance demands of wrestling. While the cycling-based protocol provided a controlled and reproducible measure of high-intensity anaerobic performance, caution is warranted when generalizing these findings to wrestling-specific movements. Future research should consider employing stabilized mounting systems, screening for caffeine habituation, and utilizing sport-specific testing protocols to further isolate and validate the ergogenic effects of these ingredients.

## 5 Conclusions

The findings of this study suggest that consuming an energy drink containing caffeine, anserine, and elderberry may enhance total power output and attenuate the exercise-induced decline in cognitive performance during high-intensity exercise, offering distinct advantages over caffeine alone. These findings suggest that a multi-ingredient energy drink may serve as an effective nutritional strategy for athletes to optimize physical output while mitigating cognitive fatigue during competition.

## Data Availability

The original contributions presented in the study are included in the article/supplementary material, further inquiries can be directed to the corresponding author.
